# A commentary on: Affective coding: the emotional dimension of agency

**DOI:** 10.3389/fnhum.2015.00142

**Published:** 2015-03-17

**Authors:** David Smailes, Peter Moseley, Sam Wilkinson

**Affiliations:** ^1^Department of Psychology, Durham UniversityDurham, UK; ^2^Department of Philosophy, Durham UniversityDurham, UK

**Keywords:** affect, emotion, agency, hallucinations, voice hearing, psychosis

We welcome Gentsch and Synofzik's (2014) review of the role of affect in modulating a person's sense of agency (SoA). The review is timely and synthesizes a great deal of evidence. However, we feel that their claims concerning the role of affect in modulating a person's SoA could be usefully extended to the study of auditory hallucinations (AH), one unusual experience not discussed in their review. In this commentary, we describe recent findings that suggest that one way in which negative affect plays a role in the development of AH is by reducing the SoA associated with self-generated cognitions and suggest that the insula may play an important role in mediating the effect of affect on SoA.

Cognitive models of AH (e.g., Bentall, 1990; Waters et al., 2012) suggest that they occur when a cognition is misattributed to an external, non-self-source. Consistent with this claim, people who experience AH tend to show a bias toward accepting the presence of a verbal stimulus on tasks designed to measure reality discrimination (i.e., they tend to confuse internal, self-generated events for external, non-self-generated events, but not vice versa; Brookwell et al., 2013). This is often demonstrated using an auditory signal detection task (SDT), in which participants must try to detect speech in an ambiguous auditory stimulus (typically white noise). On trials where the speech is absent, participants have the opportunity to make a false alarm; that is, to report that speech was present in the white noise, when it was not. Presumably, when participants make a false alarm, they have mistaken their internal representation of the speech for the externally presented, “real” speech.

Two recent studies have demonstrated a causal relation between increases in negative affect and weaker reality discrimination. Smailes et al. (2014) reported that participants who performed an auditory SDT after completing a negative mood induction (recalling an unpleasant autobiographical memory) made more false alarms, but not more hits, than did participants who performed the task after completing a neutral mood induction. Similarly, Hoskin et al. (2014), despite employing a different mood induction and a modified SDT, reported that participants were more likely to make false alarms during a condition in which they were exposed to a stressor than during a control condition. Thus, both studies showed that when participants experienced negative affect, they were more likely to misattribute internal, self-generated cognitions to an external source. These findings are consistent with data from studies that have shown that negative affect tends to precede the onset of AH in the daily lives of psychosis patients (Nayani and David, 1996; Delespaul et al., 2002), and suggest that negative affect is associated with the onset of AH, at least in part, because it modulates SoA for cognition.

Gentsch and Synofzik propose that three stages of agency processing—prospective, immediate, and retrospective—exist. First person accounts (e.g., Romme et al., 2009; Scholtus and Blanke, 2012) suggest that people who experience AH do not go through a deliberative process to determine whether an unusual auditory percept was self-generated, or was a result of an external, non-self agent. Instead, an AH is experienced “in the moment” as something that was not self-generated. Thus, in terms of Gentsch and Synofzik's proposed stages, it seems likely that the effect of negative emotions on a person's reality discrimination abilities would correspond to either prospective or immediate affective coding, rather than retrospective affective coding. That is, negative affect may reduce a person's SoA over cognition by interfering with action planning or with the generation of an accurate sensory outcome representation of a cognition. The most prominent cognitive models of AH suggest that a forward model system acts to predict the sensory outcomes of motor commands, and that dysfunction at one of a number of comparators can lead to a lack of agency over self-generated actions (Jones and Fernyhough, 2007). Interference with either action planning or accurate prediction could therefore lead to external misattributions of self-generated processes. A potential avenue for research would be to investigate whether negative affect can modulate, for example, the sensory attenuation which is associated with successful prediction via forward modeling.

Gentsch and Synofzik's review only briefly discusses the brain regions that may be involved in mediating the effects of negative affect on SoA. At two points, however, they cite evidence suggesting that damage to/atypical activity in the insula can lead to disorders of SoA. We concur that the insula is a good candidate for mediating the effects of negative affect on SoA. This is because, in addition to the evidence cited by Gentsch and Synofzik, a number of studies have shown (a) that different agency experiences are associated with changes in insula activity (e.g., Farrer and Frith, 2002; Farrer et al., 2003), (b) that insula activity is atypical in people who report AH (Wylie and Tregellas, 2010) and (c) that increases in negative affect are associated with changes in insula activity (Phan et al., 2002; Harrison et al., 2008). Research that examines whether negative affect brings about reductions in a person's SoA over their cognitions through modulation of activity in the insula is required.

Demonstrating that affective problems may play an important role in the development of AH is important for a number of reasons. First, affect-induced changes in the SoA a person has over their cognitions can help to explain why AH are typically not experienced constantly (this issue is sometimes raised as a problem for cognitive models of AH; Gallagher, 2004). Seconds, and perhaps more importantly, it opens up the possibility of novel therapeutic interventions. While the primary focus of such interventions may be on ameliorating the affective problems reported by people who hear voices, they may indirectly reduce the frequency of AH by preventing negative affect-induced modulations of a person's SoA over their cognitions.

## Conflict of interest statement

The authors declare that the research was conducted in the absence of any commercial or financial relationships that could be construed as a potential conflict of interest.
